# Enhancement Effect of Lemon Flower on the Flavor Quality of White Tea and Its Formation Mechanism

**DOI:** 10.3390/foods15030596

**Published:** 2026-02-06

**Authors:** Jun Wang, Yiwen Hu, Deyu Hu, Zhihong Lu, Li Xiang, Jinsong Xiang, Min Hong, Lili Ling, Yanyan Ma

**Affiliations:** 1 Citrus Research Institute, Southwest University, Chongqing 400712, China; wj1985@swu.edu.cn (J.W.); ywhu9050@163.com (Y.H.);; 2 National Citrus Engineering Research Center, Chongqing 400712, China; 3 Chongqing Wanzhou Economic Crops Development Center, Chongqing 404000, China; 4 Fengjie Navel Orange Industry Development Center, Chongqing 404600, China; 5 Fengjie Navel Orange Research Institute, Chongqing 404600, China

**Keywords:** white tea, lemon flower, flavor formation, taste compounds

## Abstract

This study involved developing a novel lemon flower-scented white tea (LT) through multiple aroma-imparting cycles, and taking an integrated approach to investigating its flavour formation mechanism. Sensory evaluation and electronic tongue analysis revealed that the LT exhibited more balanced taste characteristics, with significantly reduced bitterness and astringency, attributed to the decreased caffeine content and conversion of esterified catechins. Electronic nose and HS-SPME/GC-MS results confirmed that the LT had acquired a distinctive aroma characterised by floral and citrus notes, primarily originating from lemon flower volatiles such as methyl anthranilate and limonene. Multivariate statistical analysis identified 32 key differential compounds (variable importance in projection value > 1), with methyl anthranilate, β-ionone, and geraniol (relative odour activity value > 80) jointly forming the shared flavour base among teas. These findings demonstrate that lemon flower infusion can effectively enhance the sensory quality of white tea and provide theoretical support for the development of diverse floral teas.

## 1. Introduction

Edible flowers have garnered significant attention within the food industry as natural flavoring agents and functional ingredients, attributed to their distinctive aromas and bioactive compounds [1,2]. The flowers of lemon (*Citrus limon* (L.) *Osbeck*) were particularly rich in volatile terpenoids, such as D-limonene, sabinene, and *β*-pinene, which contribute to a fresh and pleasant citrus-floral aroma [3]. Although lemon flowers (LFs) have been predominantly employed in high-value perfumery and essential oil extraction [4,5], their potential as natural flavor enhancers in tea processing remains largely unexplored. White tea (WT), acknowledged as one of the six principal tea categories in China, is characterized by its minimal processing, which primarily involves withering and drying [6,7]. This gentle processing methodology aids in preserving substantial levels of bioactive compounds, including tea polyphenols, theanine, flavonoids, caffeine, and soluble sugars. These constituents collectively contribute to WT’s distinctively sweet, mellow, and subtly fragrant profile, as well as its documented health benefits, such as antioxidant and anti-inflammatory properties [8,9,10]. Nevertheless, the same minimal processing results in a delicate and subtle flavor profile, which may limit its appeal in a contemporary market that favors diverse and engaging sensory experiences. To expand its sensory appeal, the technique of flower scenting has been employed as an effective strategy to diversify and enhance the aromatic and taste dimensions of tea.

The process of flower scenting represents a crucial advanced technique in tea production, characterized by the interaction between volatile aromatic compounds emitted by flowers and the tea leaf matrix. This interaction occurs through physical adsorption and potential chemical transformations, culminating in a harmonious, multi-layered composite aroma profile. For instance, in jasmine green tea, more than 30 volatile compounds, predominantly *β*-ionone, *β*-linalool, indole, and methyl anthranilate, increase during the scenting process, while certain substances undergo heat-induced desorption in the scented green tea [11]. The dynamic equilibrium between volatile absorption and desorption collectively determines the ultimate aromatic retention and fragrance of the tea [11,12,13]. Beyond jasmine green tea, there is growing interest in utilizing various tea types as bases for jasmine tea. For example, the primary aroma compounds in jasmine black tea, such as linalool, geraniol, benzyl acetate, and methyl anthranilate, contribute floral, sweet, and green notes to the infusion [14]. These distinctions from jasmine green tea indicate that the choice of tea base significantly influences the adsorption and retention of floral aromas.

The scenting process played a crucial role in shaping both the aroma and taste profiles of tea. Previous studies have demonstrated that enzymatic and non-enzymatic reactions during scenting could modify the composition and proportion of non-volatile compounds, including catechins, flavonoid glycosides, and amino acids. These alterations influence the intensity and balance of bitterness, astringency, umami, and sweetness [3]. For instance, the transformation of non-volatile components such as tea polyphenols and esterified catechins during scenting was found to shift the taste profile of green tea from strongly astringent to mellow and sweet. Further studies, such as those conducted by [15], have demonstrated that post-scenting, the caffeine content decreases significantly, while the concentration of methyl anthranilate doubles. In the case of dendrobium officinale flower tea, the polyphenol content decreases markedly after scenting, whereas the amino acid content increases substantially [16]. Additionally, the hydrolysis of galloylated catechins was shown to reduce astringency while enhancing sweetness [17].

Nevertheless, current research remains predominantly focused on traditional floral varieties such as jasmine and osmanthus. In recent years, citrus-based teas, often processed with citrus peels or blossoms, have gained popularity, highlighting the potential of citrus flowers as scenting agents. Among various citrus blossoms, the lemon flower is distinguished by its exceptionally high limonene content [18]. However, LFs, which produce over 60% of its annual blooms in spring while maintaining a remarkably low fruit set rate of only 1–2%, represent a notably underutilized aromatic resource. Field observations indicate that over 60% of lemon flowers undergo natural abscission during the flowering, resulting in substantial resource wastage. The integration of this novel scenting material with WT thus offers a promising approach to enhance the utilization efficiency of this agricultural by-product, although the underlying flavor formation mechanisms require systematic investigation.

Given the high cost of aged white tea and its limited value for secondary processing, this study innovatively employs newly processed white tea as the base material. This study represents a pioneering effort in the systematic application of LF scenting in WT, utilizing a multimodal analytical approach to thoroughly elucidate its flavor chemistry. Specifically, the research objectives were to: (1) objectively quantify the evolution of aroma and taste profiles in lemon flower-scented white tea (LT) through a combination of professional sensory evaluation and electronic tongue/nose technology; (2) accurately identify changes in the composition of volatile flavor compounds using headspace-solid-phase microextraction coupled with gas chromatography-mass spectrometry (HS-SPME-GC-MS), and screen key aromatic active components based on criteria such as variable importance projection and odor activity values; and (3) integrate non-volatile component analysis to investigate the material basis and formation mechanisms of LF scenting in shaping WT’s distinctive flavor profile. This study aims to provide a robust theoretical foundation and practical guidance for the standardized production and flavor-oriented process optimization of high-quality LT products.

## 2. Materials and Methods

### 2.1. Sample Preparation and Collection

**Figure 1:**
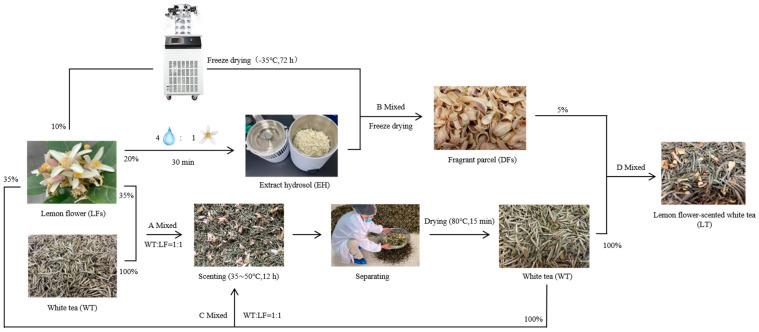
Processing flowchart of lemon flower-scented white tea.

Fresh leaf flowers (LFs) were harvested in February 2025 from the germplasm resource nursery at the Citrus Research Institute, Southwest University, Chongqing, China. Following the harvest, the flowers were meticulously sorted to eliminate impurities and were immediately stored at −80 °C for subsequent use in scenting experiments. The WT utilized in this study was commercially procured from Dajin, Kaizhou, Chongqing, China, and was processed for scenting upon arrival. The overall scenting procedure is depicted in Figure 1. Specifically, the LFs were allocated according to the proportions illustrated in Figure 1. (1) Initially, 10% of the LFs were freeze-dried at −35 °C for 72 h. (2) Subsequently, 20% of the LFs were combined with four times their weight of purified water and extracted for 30 min to yield an essential oil hydrosol (EH). This resulting EH mixture was uniformly applied to the freeze-dried petals, which were then subjected to an additional freeze-drying process until the moisture content of the dried petals (DFs) fell below 6%. (3) Furthermore, 35% of the LFs were blended with WT leaves (initial moisture content of 6%) at a 1:1 weight ratio. (4) Similarly, 35% of the LFs were mixed with previously scented WT leaves at a weight ratio of 1:1 and underwent the scenting process again. Scenting process: The mixture underwent two rounds of scenting at temperatures ranging from 35 °C to 50 °C, with each round lasting 12 h. Following the scenting process, the used LFs were discarded. The scented WT underwent a two-step drying procedure: Initially at 85 °C for 10 min to achieve a moisture content of 8%, and subsequently at 80 °C for 6 min to stabilize the final moisture content at approximately 6%. Ultimately, the twice-dried WT was combined with the DFs at a ratio of 5% (*w*/*w*), then packaged in waterproof and moisture-proof bags, sealed, and stored at 4 °C for subsequent use.

### 2.2. Sensory Evaluation

The sensory quality of LT and WT was assessed in accordance with China’s national standard (GB/T 23776-2018 [19]). A professional panel consisting of 10 trained evaluators, equally divided by gender (five females and five males, aged 30–55 years, all of whom hold national professional qualification certificates), conducted the evaluation. For each sample, 3 g of tea leaves were infused in 150 mL of boiling water at 100 °C for a duration of precisely 5 min. Post-infusion, the liquor was separated from the leaves for evaluation. Sensory attributes were rated on a 100-point scale, with specific weightings assigned as follows: 20% for appearance, 5% for liquor color, 35% for aroma, 30% for taste, and 10% for the appearance of the infused leaves. All assessors provided informed consent and underwent appropriate training prior to the evaluation sessions. The final score for each sample was calculated as the average of all panelists’ ratings.

### 2.3. Electronic Tongue Analysis

The taste profiles of the tea samples were analyzed and evaluated utilizing a TS-5000Z Taste-Sensing System (Intelligent Sensor Technology Co., Ltd., Kanagawa, Japan). This instrument was equipped with a sensor array comprising six lipid/polymer membrane sensors (AAE, CT0, CA0, C00, AE1, and GL1), which collectively assessed nine taste attributes: sourness, bitterness, astringency, sweetness, umami, richness, saltiness, aftertaste-A (astringency), and aftertaste-B (bitterness).

### 2.4. Electronic Nose Data Collection and Analysis

The volatile compounds present in LT and WT were analyzed utilizing a PEN3 portable electronic nose (WNA Airsense Analysentechnik GmbH, Mecklenburg, Germany), which is equipped with an array of ten metal oxide semiconductor sensors. For each experimental sample, 4 g of tea leaves were infused with 150 mL of boiling water (100 °C) for a duration of four minutes. Subsequently, the infusion was filtered and allowed to cool to room temperature, after which 5 mL were transferred to a 100 mL beaker. The beaker was sealed with a double-layer of preservative film and equilibrated for one hour to facilitate headspace formation. Measurements were conducted under specified conditions: an injection flow rate of 400 mL/min, a sensor flushing time of 80 s, and a data acquisition time of 80 s. Sensor response data recorded between 75 and 77 s were averaged for subsequent analysis.

### 2.5. Determination of Taste Compounds

The determination of total free amino acids was conducted according to the Chinese National Standard GB/T 8314-2013 [20]. Caffeine content was analyzed following the method specified in GB/T 8312-2013 [21]. The composition of catechins was determined in accordance with GB/T 8313-2018 [22]. Theanine content was measured using the high-performance liquid chromatography (HPLC) method described in GB/T 23193-2017 [23]. Soluble sugars were quantified by the anthrone reagent method.

The concentrations of gallic acid (GA) in the samples were concurrently quantified utilizing a 1100 HPLC system (Agilent Technology, Santa Clara, CA, USA). To prepare the samples, approximately 0.3 g was precisely weighed and placed into a 15 mL centrifuge tube. Subsequently, 10 mL of 50% methanol was added, and the mixture was agitated on an oscillator for 5 min. The samples were then sonicated at room temperature for 30 min and centrifuged at 4000 rpm for 10 min. The supernatant was transferred to a new centrifuge tube, and an additional 10 mL of 50% methanol was added to the residue. This mixture was thoroughly combined and allowed to stand at 4 °C for 1 h. Following extraction, the samples were sonicated for another 30 min and centrifuged. The two supernatants were combined, diluted to a final volume of 20 mL, mixed thoroughly, and filtered through a 0.22 μm membrane filter prior to liquid chromatography analysis. The chromatographic conditions were as follows: an ODS-100V C18 column (4.6 mm × 250 mm, 5 μm) was employed, with UV detection at 280 nm. Mobile phase A was prepared by combining 90 mL of acetonitrile, 20 mL of acetic acid, and 2 mL of EDTA solution (10 mg/mL) in a 1 L volumetric flask. The mixture was then diluted to volume with ultrapure water and filtered through a 0.45 μm membrane. Mobile phase B was prepared similarly using 800 mL of acetonitrile, 20 mL of acetic acid, and 2 mL of EDTA solution (10 mg/mL), followed by dilution to the mark with ultrapure water and filtration. The mobile phase flow rate was maintained at 1.0 mL/min. The column temperature was kept constant at 35 °C, and the injection volume was set at 20 μL. Gradient elution was performed according to the following program: 0–10 min, 100% A; 10–25 min, 75% A and 25% B; 25–26 min, 100% A; and 26–30 min, 100% A for re-equilibration. Retention times and signal intensities of the target compounds in each sample are provided in Table S1.

### 2.6. Volatile Compound Analysis

#### 2.6.1. Headspace Solid-Phase Microextraction (HS-SPME) Procedure

Agilent 8890-7000E system equipped with an Agilent DB-WAX capillary column (30 m × 0.25 mm, 0.25 μm) was used for the analysis of volatile compounds in samples. In a nutshell, 0.5 g of a tea sample was infused with 5 mL of boiling water and transferred into 20 mL headspace injection vial with 1.5 μL of a 14.25 μg/mL cyclohexanone solution as the internal standard. A 120 μm DVB/CAR-WR/PDMS fiber (Agilent Technologies, USA) was used to extract volatile compounds from the headspace vial for 50 min at 60 °C after equilibration for 10 min. After that, the fiber was immediately inserted into the gas chromatograph injector installed on a column and desorbed at 225 °C for 5 min. High purity helium (over 99.999%) was used as the carrier gas with a constant flow rate of 1 mL/min.

The analysis of volatile compounds in the samples was conducted using an Agilent 8890-7000E system, which was equipped with an Agilent DB-WAX capillary column (30 m × 0.25 mm, 0.25 μm). Specifically, 0.5 g of a tea sample was infused with 5 mL of boiling water and subsequently transferred into a 20 mL headspace injection vial, along with 1.5 μL of a 14.25 μg/mL cyclohexanone solution serving as the internal standard. A 120 μm DVB/CAR-WR/PDMS fiber (Agilent Technologies, USA) was employed to extract volatile compounds from the headspace vial over a period of 50 min at 60 °C, following a 10 min equilibration phase. Subsequently, the fiber was promptly inserted into the gas chromatograph injector, which was installed on the column, and desorbed at 225 °C for 5 min. High-purity helium (exceeding 99.999%) was utilized as the carrier gas, maintaining a constant flow rate of 1 mL/min.

#### 2.6.2. GC-MS Conditions

The oven temperature was programmed according to the following protocol: initially held at 40 °C for 2 min, then increased to 85 °C at a rate of 3 °C per minute and maintained for 2 min. Subsequently, the temperature was raised to 110 °C at a rate of 2 °C per minute and held for 2 min, followed by an increase to 160 °C at 5 °C per minute, maintained for 1 min. The temperature was further increased to 225 °C at 5 °C per minute and held for 5 min, and finally increased to 230 °C at 10 °C per minute, maintained for 8 min. Mass spectrometry analysis was performed using electron ionization at 70 eV, with a scan range of *m*/*z* 50–650. Each treatment group underwent three replicate trials.

#### 2.6.3. Qualitative and Quantitative Analyses of Volatile Compounds

Volatile compounds were analyzed by the identification and quantification procedure. The retention indices were calculated for each detected peak by C_5_-C_25_ n-alkane standards (Shanghai Anpu Experimental Technology Co., Ltd., Shanghai, China). Tentative identification was achieved by matching both the mass spectra and RIs against reference data in the NIST 20 library. Subsequently, quantification of identified volatile compounds was performed using the internal (cyclohexanone).

The raw chromatographic peak areas of all target analytes were normalized to the peak area of the internal standard (cyclohexanone) to account for potential variations associated with sample preparation and instrumental analysis. Blank controls were incorporated into the GC-MS autosampler sequence to monitor and correct for background interference.

#### 2.6.4. Relative Odor Activity Values (rOAVs)

The relative odor activity value (rOAV) was calculated as the ratio of the concentration of each volatile compound in the sample to its odor threshold in water. Compounds with an rOAV greater than 1 were typically considered to significantly contribute to the overall aroma of the analyte sample. The odor thresholds used in this study were sourced from pertinent literature. The formula for this calculation was as follows: ROAVi ≈ C_i_/T_i_ × T_max_/C_max_ × 100, where C_i_ denotes the relative concentration (μg/kg) of volatile organic compounds (VOCs), T_i_ represents the threshold concentration (μg/kg) of the compound in water, and C_max_ and T_max_ refer to the relative content and threshold of the compounds that most significantly influence the overall flavor.

### 2.7. Statistical Analysis

Each sample underwent triplicate parallel measurements. Principal component analysis (PCA) and orthogonal partial least squares discriminant analysis (OPLS-DA) were conducted using SIMCA-P 14.1 software (Uppsala, Sweden). Volatile compounds with variable importance for projection (VIP) values exceeding 1 were identified. A *p*-value of less than 0.05 was considered to indicate statistical significance. The heatmap and additional visualizations were generated using Origin 2025 software (OriginLab Co., Northampton, MA, USA).

## 3. Results and Discussion

### 3.1. Sensory Evaluation of WT and LT

**Figure 2:**
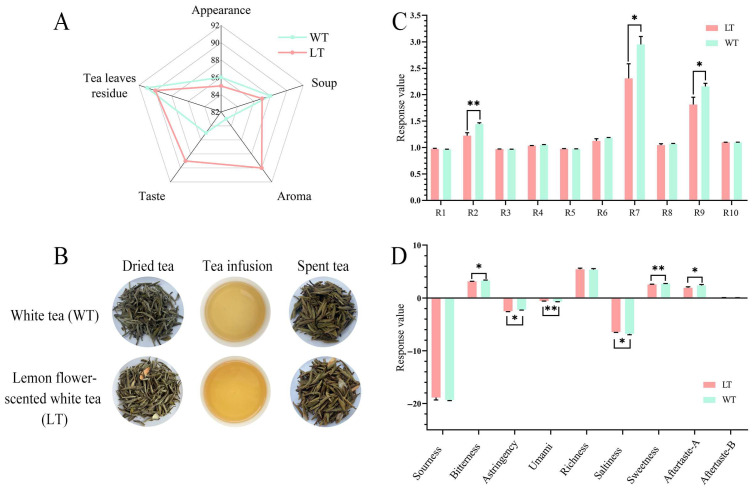
Comparisons of appearance, aroma, and taste between WT and LT. Sensory evaluation radar charts (**A**) and Visual appearances of dried tea leaves, tea infusions, and spent tea leaves (**B**). Bar chart representing response values of electronic tongue (**C**) and electronic nose (**D**) analysis results. * represents *p* < 0.05, ** represents *p* < 0.01, represents *p* < 0.001.

The results of the sensory evaluation are presented in Figure 2A. The overall sensory score for the LT was significantly higher than that for the WT, with scores of 88.55 and 85.25, respectively. Although the WT sample received marginally higher scores for appearance, liquor color, and infused leaf evaluation, these differences did not reach statistical significance. Detailed sensory data are provided in Table S2. In terms of infusion colour, both samples displayed a light yellow hue with a bright appearance. However, the LT sample showed a marginally deeper hue than the WT sample (Figure 2B). This difference may be attributed to enzymatic reactions during processing that enhance the pigment content of the tea leaves [24]. The most pronounced sensory differences between LT and WT were observed in aroma and taste, with aroma serving as the most distinguishing attribute. The infusions of both teas exhibited a fresh, sweet, and brisk taste profile. Notably, when brewed at high temperature, WT displayed a distinct grassy note, whereas in LT, this characteristic was modified into a softer, more harmonious profile, yielding a more layered and complex flavor enriched with floral nuances. To quantitatively characterize these sensory variations, further analyses were conducted using an electronic tongue and an electronic nose system.

### 3.2. Taste Profile Evolution Revealed by E-Tongue

To objectively characterize the differences in taste quality between LT and WT, an electronic tongue evaluation was conducted. As illustrated in Figure 2C, significant differences were observed in the majority of taste attributes between the two samples. No significant differences were detected in sourness, richness, or aftertaste-B. However, LT demonstrated significantly lower levels of bitterness, astringency, sweetness, and aftertaste-A than WT, while umami and saltiness were enhanced according to instrumental (e-tongue) analysis. It was noteworthy that the increase in instrumental umami signal did not correspond to a sensorially distinguishable difference, as the umami intensity of LT remained below tasteless. These findings collectively suggest that LFs scenting effectively rebalances the taste profile of white tea by suppressing bitterness, astringency, and sweetness, and by modulating salty and umami-related signatures.

### 3.3. Aroma Profile Reorganization Captured by E-Nose

The flavor profiles of LT and WT were systematically analyzed using an electronic nose system. As illustrated in Figure 2D, the distinctions between the two tea varieties were predominantly detected by sensors R2, R7, and R9. Notably, LT demonstrated slightly higher response values than WT on sensors R1, R3, R5, and R10, which were primarily sensitive to aromatic compounds such as benzene derivatives and alkanes. In contrast, LT exhibited significantly lower responses on the remaining sensors, particularly R2, R7, and R9, which primarily detect inorganic sulfides, organic sulfur compounds, and nitrogen oxides. Certain sulfur-containing volatiles in tea have been well-documented as contributors to undesirable off-odors [25]. The substantially lower signals of LT on these sensors indicate that the lemon flower scenting process effectively alters the overall aroma profile of WT. The introduction of citrus floral volatiles may mask or transform certain compounds, thereby conferring a more balanced and pleasant aromatic profile to LT.

### 3.4. Dynamic Changes in Non-Volatile Taste Compounds

#### 3.4.1. Bitterness and Astringency

**Figure 3:**
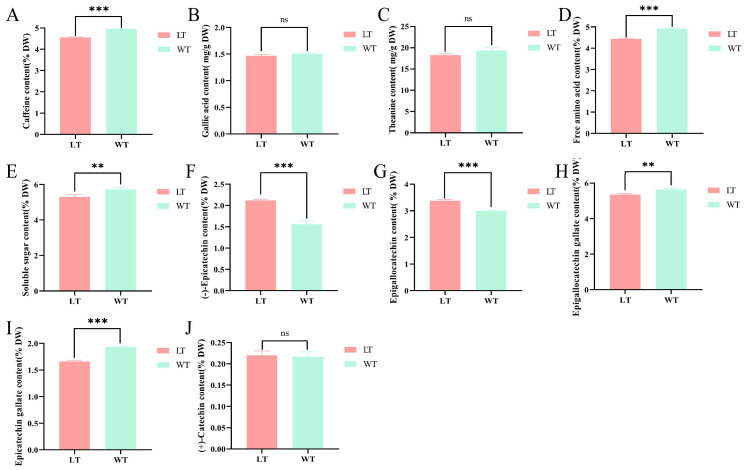
The differences in key flavor substances between WT and LT. (**A**) Caffeine, (**B**) Gallic acid, (**C**) Theanine, (**D**) Free amino acid, (**E**) Soluble sugar, (**F**) (−)-Epicatechin, (**G**) Epigallocatechin, (**H**) Epigallocatechin gallate, (**I**) (+)-Catechin, (**J**) Epicatechin gallcte. represents *p* < 0.05, ** represents *p* < 0.01, *** represents *p* < 0.001.

Significant differences in bitterness and astringency were identified between WT and LT through electronic tongue analysis and sensory evaluation. Bitterness and astringency frequently demonstrate synergistic interactions, as numerous bitter compounds also enhance astringency [26]. Caffeine, the predominant alkaloid in tea, was identified as a principal contributor to both bitterness and astringency, while catechins and gallic acid (GA) were recognized as major polyphenolic constituents influencing these sensory characteristics [27]. Consequently, the concentrations of these compounds were quantified, and the findings are depicted in Figure 3. Upon exposure to LF scenting, the caffeine and GA content in LT exhibited a decrease to varying extents, whereas the total catechin content increased significantly compared to WT. Specifically, the caffeine content in LT was measured at 4.55%, a statistically significant reduction compared to WT. Conversely, no significant difference was observed in the GA content, which remained at 1.47% for both LT and WT.

Additionally, five catechins were quantified: (−)-epicatechin (EC), epigallocatechin (EGC), epigallocatechin gallate (EGCG), (+)-catechin, and epicatechin gallate (ECG). The findings indicated that the EC and EGC contents in LT increased significantly by 35.61% and 12.56%, respectively, whereas the EGCG and ECG contents decreased by 5.89% and 13.97%, respectively. The content of (+)-catechin remained constant at 0.22% in both LT and WT. Epigallocatechin gallate (EGCG), a predominant polyphenol in tea, has been documented to undergo degradation during thermal processing due to processes such as isomerization, degradation, and polymerization, which demonstrated that EGCG experiences isomerization and degradation at baking temperatures ranging from 80 °C to 150 °C [28,29], resulting in the formation of gallocatechin gallate (GCG), epicatechin gallate (ECG), epicatechin (EC), and gallocatechin (GC), which collectively contribute to a marked reduction in EGCG content.

#### 3.4.2. Umami and Sweetness

Free amino acids (FAAs) have been identified as critical components contributing to the refreshing taste and umami sensation in tea, with their concentration showing a positive correlation with taste intensity [30]. Theanine, in particular, plays a significant role due to its strong association with the umami characteristics of tea. This study determined that the theanine content in WT was 19.37 g/kg, whereas it decreased to 18.25 g/kg in LT, indicating a reduction of 5.78%. Similarly, the total free amino acid content declined from 4.93% in WT to 4.44% in LT. This reduction was attributed to the multiple scenting cycles involved in LT processing, each followed by drying at 80 °C for 15 min. Such thermal treatment likely compromised amino acid stability, with the high-temperature drying step contributing to reduced amino acid levels. This was consistent with previous findings that lower brewing temperatures better preserve amino acid composition and enhance the umami taste of tea infusions [31]. Furthermore, under high-temperature conditions, epigallocatechin gallate (EGCG) was found to undergo nucleophilic reactions with theanine, leading to the formation of N-ethyl-2-pyrrolidinone-substituted flavan-3-ols (EPSFs). Moreover, L-theanine was susceptible to thermal degradation, resulting in the formation of compounds such as N-ethylformamide, ethylamine, and 2-pyrrolidinone. It also had the potential to react with soluble sugars, leading to the production of heterocyclic compounds, including N-ethylpyrrolidone and pyrazines [32].

Soluble sugars have been identified as a crucial component contributing to the sweetness of tea infusions [33,34]. In the present study, the soluble sugar content in LT was quantified at 5.31%, representing a 0.33% decrease relative to WT. This reduction was likely due to caramelization and Maillard reactions occurring between amino acids and reducing sugars during the high-temperature drying process. These complex reactions consume soluble sugars and amino acids while generating various non-volatile pigment compounds [34,35]. These chemical transformations not only account for the reduction in taste-active components but also correlate with the observed darkening of the LT infusion color (Figure 2B).

### 3.5. Analysis of Volatile Compounds in WT and LT

#### 3.5.1. Composition and Comparative Analysis of VOCs

**Figure 4:**
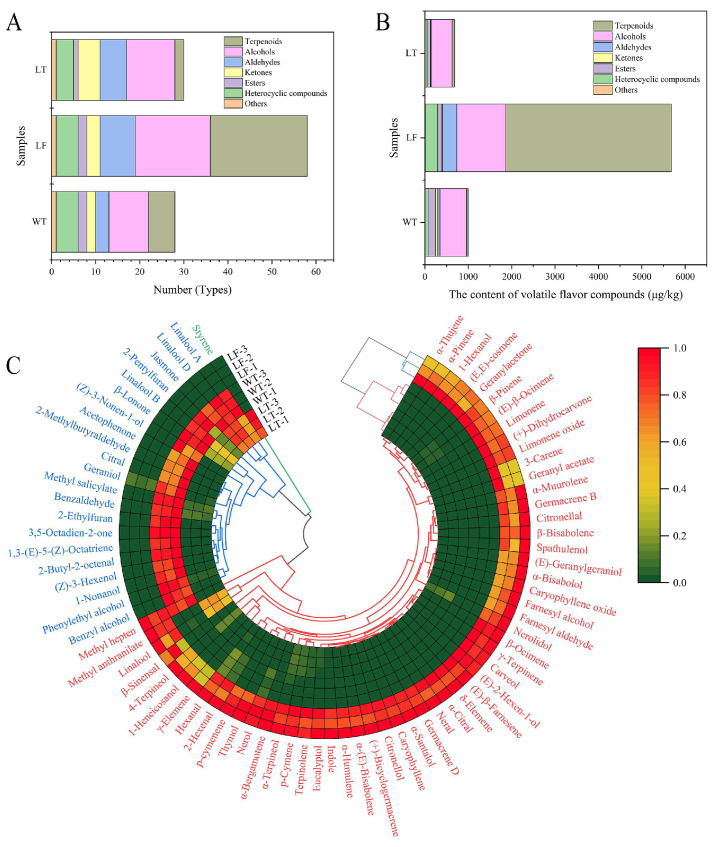
Volatile compound profiles of LF, WT, and LT. (**A**) The number of volatile compounds identified in each sample. (**B**) The average relative content of each volatile compound in each category. (**C**) A heatmap of 78 differential volatile compounds between LF, WT, and LT.

**Figure 5:**
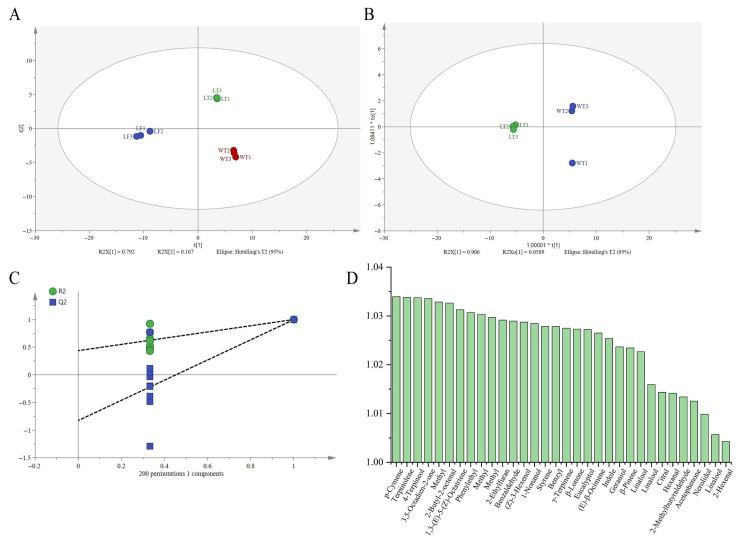
Comparative analysis of volatile components of LT and WT. (**A**) PCA of these volatile compound profiles. (**B**) OPLS-DA score plot. (**C**) Permutation tests plot. (**D**) VIP plot.

Incorporating LFs into the HS-SPME/GC-MS analysis enabled a more precise tracing of the origins of the characteristic volatile compounds in LT and elucidated the modifications to the flavor profile induced by the scenting process. A semi-quantitative determination of volatile compounds in LFs, WT, and LT was conducted using cyclohexanone as an internal standard. The concentrations of all identified compounds are detailed in Table S3. A comprehensive analysis of the volatile organic compound (VOC) profiles identified 78 VOCs, comprising 22 terpenoids, 22 alcohols, 11 aldehydes, seven ketones, three esters, 10 heterocyclic compounds, and three other compounds. Distinct differences in VOC composition were observed among the samples, with 28, 58, and 30 compounds detected in LT, LF, and WT, respectively (Figure 4A). Significant variations in the types and contents of volatile flavor compounds were evident among the three tea infusions (Figure 5A). Principal component analysis (PCA) effectively discriminated between the three sample groups, and the score plot revealed clear separation trends. This distinct clustering pattern indicated fundamental differences in the volatile compound profiles, reflecting their characteristic flavour profiles.

Terpenoids were the predominant volatile compounds in LF, comprising 67.21% of the total volatile content, as illustrated in Figure 4B,C. Limonene was detected at the highest concentration (1816.77 μg/kg) in LF, imparting a fresh lemon aroma and serving as a key contributor to its characteristic lemon flavor [36]. In contrast, only two terpenoids, namely limonene and *β*-ocimene, were identified in WT, both present at minimal concentrations. Following the scenting process, the variety and concentration of terpenoids in LT increased, with six terpenoids being identified. The total terpenoid content increased from 33.81 μg/kg in WT to 57.43 μg/kg in LT, primarily due to elevated levels of limonene, γ-terpinene, and *β*-pinene.

In the context of volatile flavor compounds, alcohols constitute a significant group in LF, ranking second only to terpenoids in terms of both abundance and diversity. In WT, alcohols emerge as the most prevalent volatile compounds, with a total concentration of 607.13 μg/kg (Figure 4B,C). The predominant alcohols in WT were linalool, geraniol, and phenethyl alcohol, which together account for 91.89% of the total alcohol content. Linalool contributed a floral aroma as a key scent component of pomelo flowers, while geraniol imparted a sweet, rosy note with subtle fruity undertones. Together, the two compounds enhanced the overall fruity and citrus flavor profile. LF demonstrates a greater diversity of alcohols, with 17 different alcohols identified, amounting to a total of 1126.14 μg/kg. Among these, linalool, nerolidol, and eucalyptol were present at relatively high concentrations. Conversely, LT exhibits the lowest total alcohol content among the three samples, with a concentration of 476.56 μg/kg. In comparison to LF and WT, the majority of alcoholic compounds in LT show a decrease to varying extents.

As a lightly fermented tea, white tea was characterized by aldehydes that predominantly contribute fresh, green, and grassy aromas similar to those found in green tea [37]. In LT, aldehydes constituted the third-most abundant category of volatile flavor compounds, with eight distinct compounds identified, amounting to a total of 329.71 μg/kg (Figure 4B,C). In WT, the concentration of citral was measured at 5.48 μg/kg. Although this concentration was lower than those of benzaldehyde and 2-butyl-2-octenal, citral’s low sensory threshold of 0.04 μg/kg renders it a significant contributor to the distinctive lemon-like freshness and citrusy coolness characteristic of white tea [38].

Among the six primary categories of flavor compounds identified, ketones and esters were the only classes exhibiting higher total concentrations in WT compared to LF samples. The majority of ketones and esters were uniquely present in either WT or LF, with only negligible quantities retained in LT after the scenting process (refer to Figure 4B,C). The concentration of ketones experienced a substantial decline, from 53.42 μg/kg in WT to 6.55 μg/kg in LT, representing a reduction of 87.74%. Similarly, methyl salicylate, which imparts wintergreen and minty aromas, was the predominant ester in WT (as reported by Chen [39] and Xie [40]) and exhibited a marked decrease from 167.18 μg/kg in WT to 18.25 μg/kg in LT, corresponding to a reduction of 89.08%. Conversely, methyl anthranilate, a distinctive component of lemon flower, was absent in WT but detected in LF at a concentration of 86.54 μg/kg. Through the process of physical adsorption during scenting, its concentration in LT increased to 58.00 μg/kg.

While heterocyclic compounds were not the primary volatile constituents in lemon flower and white tea, they nonetheless played significant roles in defining their floral and tea-like characteristics. Indole emerged as the most prevalent heterocyclic compound in LF but was absent in WT (Figure 4C). Following the scenting of lemon flower, the indole content in LT increased to 2.59 μg/kg, imparting a sweet floral aroma [41]. Among the heterocyclic compounds present in WT, linalool oxides (including linalool oxide A, B, and D) were predominant. These oxides persisted in LT, contributing to its pleasant floral notes [42]. Various types of linalool oxides have also been identified in Yunnan white tea and were considered crucial markers for differentiating between Fujian and Yunnan white teas [43].

#### 3.5.2. Screening of Key Volatile Flavor Compounds

The formation of volatile flavor compounds plays a crucial role in determining the distinctive flavor profile of LT, with the perceived intensity being influenced by both the concentration of these compounds and their odor thresholds. To examine the effect of lemon flower scenting on the aroma profile of WT and to identify key volatile markers, orthogonal partial least-squares discriminant analysis (OPLS-DA) was employed to compare LT and WT. The developed OPLS-DA model (Figure 5B) exhibited high reliability, with an R^2^X value of 0.965, indicating that the two principal components accounted for 96.5% of the variance in the X-variables. Additionally, an R^2^Y value of 0.999 demonstrated that the model explained 99.9% of the variance in the Y-variables, while a Q^2^ value of 0.996 reflected a predictive capability of 99.6%. A permutation test with 200 iterations confirmed the model’s stability and the absence of overfitting (Figure 5C). In conclusion, the constructed OPLS-DA model proved to be highly effective in this study. Based on a variable importance in projection (VIP) value greater than 1, 32 potential markers were initially identified as differentially expressed compounds (Figure 5D).

Nevertheless, the concentration of volatile compounds alone proved inadequate for accurately evaluating their contribution to the aroma quality of LT [13]. Consequently, this study employed the relative odour activity value (rOAV) to precisely identify the key aroma-active compounds that define LT’s distinctive scent. As illustrated in Figure 5D and Table 1, ten compounds were identified in LT and eight in WT, based on VIP and rOAV criteria (VIP > 1, *p* < 0.05, and rOAV > 1). Methyl anthranilate, with an rOAV of 100, was the compound with the highest rOAV in LT, imparting floral, honey, and peach aromas [13]. In contrast, the most influential compound in WT was 3,5-octadien-2-one, also with an rOAV of 100. Both *β*-ionone and geraniol exhibited high rOAV values in LT and WT, underscoring their significant role in the characteristic flavour profile of both tea types [44]. Notably, *β*-ionone has also been identified as contributing to the essential floral and sweet characters of the white teas and green teas [45,46]. Additionally, linalool oxides A and B made significant contributions to the overall aroma of LT.

## 4. Discussion

### 4.1. Flavor Modulation Through Lemon Flower Scenting: From Sensory Perception to Chemical Basis

Sensory evaluation serves as the most direct measure of tea quality. In this study, LT achieved a significantly higher overall sensory score compared to the base (WT), with aroma identified as the most distinguishing attribute. This enhancement was quantitatively corroborated by intelligent sensory technologies. The electronic tongue analysis revealed that LT exhibited notably reduced bitterness, astringency, and sweetness alongside significantly enhanced umami and saltiness. Concurrently, the electronic nose data indicated lemon flower had influenced the aroma of white tea. Although human sensory evaluation captures complex perceptual interactions, albeit with inherent subjectivity, electronic tongue and nose systems offer objective, quantifiable measures of flavor and aroma characteristics. Together, these complementary approaches suggest that the scenting process not only introduces new aromas but may also mitigate or mask certain negative odorants present in the base tea, leading to a more refined overall aroma.

The chemical basis for the altered taste profile was rooted in the dynamic changes in key non-volatile compounds. The significant reduction in bitterness and astringency in LT can be primarily attributed to two synergistic transformations. First, the reduction in caffeine content may be explained by two potential mechanisms: (1) the formation of complexes between caffeine and the abundant catechins present in LFs [47], and (2) the physical adsorption and subsequent transfer of caffeine from the tea leaves to the LFs during the scenting process [48]. More critically, we observed a marked compositional shift within the catechin pool. The content of astringent and bitter esterified catechins (EGCG and ECG) decreased, while that of non-esterified catechins (EC and EGC), which impart milder bitterness, increased. This conversion, likely driven by thermal reactions during the drying phases of scenting [28,29], was a well-documented pathway for modulating tea astringency. Notably, esterified catechins, including EGCG and ECG, have been strongly associated with astringency and bitterness, whereas non-esterified catechins, such as EC and epigallocatechin (EGC), predominantly impart bitterness and exhibit reduced astringency [49]. The conversion of esterified catechins to non-esterified catechins during processing has been reported to diminish the intensity of bitterness and astringency [50]. This transformation mechanism offers a coherent chemical rationale for the observed reduction in bitterness and astringency as detected by the electronic tongue in LT samples.

An intriguing finding was the enhancement of umami in LT despite a concurrent decrease in total free amino acids (FAAs) and theanine, classical umami contributors. This apparent paradox highlights the limitation of relying solely on the concentration of known compounds. It strongly suggests the potential formation or increase in other umami-enhancing compounds during the complex scenting process. These potential compounds could include small peptides or maillard reaction derivatives formed between amino acids and sugars [51], which have been reported to exhibit potent umami characteristics [52].

### 4.2. Aroma Profile Reconstruction: Incorporation, Loss, and Synergy

The HS-SPME/GC-MS analysis provided a detailed map of the volatile compound (VOC) landscape, elucidating how LF scenting reconstructs the aroma profile of WT. The process was characterized by three simultaneous phenomena: incorporation, loss, and synergy.

Successful incorporation of characteristic notes: the scenting process successfully infused LT with key terpenoids from LF, most notably limonene, which imparts a fresh citrus character. The detection of methyl anthranilate, a signature compound of LF, in LT but not in WT, was direct evidence of successful volatile transfer. Its high rOAV value confirms its role as a core contributor to the unique floral-fruity aroma of LT.

The flavor-reducing effect: conversely, a widespread reduction in the alcohol content were observed in LT compared to both LF and WT. This “flavor-reducing effect” was common in scented tea processing and can be attributed to the evaporation of volatile compounds in the processing of flower tea, or their potential chemical transformation. This loss indicates that aroma development was not merely additive but involved selective retention and modification.

Synergistic interactions shape the complex aroma profile: the overall aroma profile of LT likely stemmed from the complex interactions among the identified volatile compounds, as well as between volatiles and the tea matrix, rather than the simple additive or reductive contributions of individual compounds. These underlying interactions played a decisive role in shaping the final olfactory character. Key compounds such as methyl anthranilate (floral-peachy), β-ionone (floral-woody), and geraniol (rosy) formed a synergistic mixture, the combined sensory impact of which exceeded that of the individual compounds, significantly enhancing the overall aromatic complexity [53,54]. The role of linalool oxides in enhancing floral notes was also noteworthy [47]. Moreover, the pronounced citrus character in LT likely resulted from synergistic interactions between limonene and complementary terpenes or oxygenated compounds. Conversely, the milder grassy note in LT compared to WT can be attributed to a masking effect, whereby potent top notes (e.g., limonene, methyl anthranilate, geraniol) suppressed the perception of underlying green alcohol. At the receptor level, such perceptual effects may involve competition for or allosteric modulation of active sites in olfactory receptors [55]. Non-covalent interactions between aroma compounds and non-volatile components in the tea matrix (e.g., polyphenols, proteins and polysaccharides), such as hydrogen bonding, hydrophobic effects, and π-π stacking, may regulate the distribution of aromas, their release kinetics, and stability during smoking and brewing processes [56]. For example, the composition and concentration of polyphenols can significantly impact the volatility of monoterpenes [57,58]. Specific phenolic acids (e.g., GA, p-coumaric acid) can also bind to aroma molecules via non-covalent interactions such as hydrogen bonding, thereby regulating the release dynamics and shaping the overall aroma profile at the molecular level [59]. The close VIP values among the top differential compounds further support this notion of a balanced, synergistic contribution from multiple volatiles rather than dominance by one. Future studies employing sensory-interaction models (e.g., σ-τ plot, U-model), molecular simulation techniques, and other advanced methodologies would help to quantify these interactions and further elucidate the mechanisms underlying the harmonious flavor of LT.

## 5. Conclusions

This study successfully developed LT with unique sensory attributes through iterative scenting processes. A multi-modal analytical approach was employed to thoroughly elucidate the effects of LF scenting on the taste and flavor profiles of WT. The scenting process significantly optimized the taste profile, which was closely associated with alterations in key taste compounds, including caffeine and catechins. In terms of flavor characteristics, the aroma profile of LT integrated the base notes of WT with distinctive volatiles from LFs. Compounds such as limonene and methyl anthranilate contributed distinct citrus and floral attributes to the tea, with the latter identified as the primary component responsible for LT’s unique floral and fruity aroma. Additionally, *β*-ionone and geraniol were instrumental in shaping the shared flavor characteristics of both tea types. The developed LT addresses the increasing consumer demand for diverse, high-quality tea beverages and establishes a methodological framework for quality-driven design and process innovation in the floral tea industry. Furthermore, the process demonstrates a practical model for agricultural by-product valorization: the collection of approximately 40 kg of thinned lemon flowers per mu (Chinese acre) can generate direct economic return for growers, while simultaneously creating a novel tea product that broadens market appeal and enhances the overall value chain of both the citrus and tea industries. Future research will aim to elucidate the dynamic migration of flavor components during the scenting process and to identify key umami-enhancing compounds, with the objective of further enhancing the quality of scented teas.

## Figures and Tables

**Table 1:** Relative odor Activity Values (rOAVs) of aroma active compounds in LT and WT.

Num.	VOCs	VIP	Odor Threshold (μg/kg)	rOAV	
				LT	WT
1	*p*-Cymene	1.03394	5.01	9.77	-
2	Methyl salicylate	1.03354	40	2.36	1.78
3	3,5-Octadien-2-one	1.03263	0.15	-	100
4	Phenylethyl alcohol	1.03069	45	0.68	1.01
5	Methyl anthranilate	1.03033	3	100	-
6	1-Nonanol	1.02847	1.1	-	2.15
7	Styrene	1.02783	3.6	4.73	-
8	Benzyl alcohol	1.02782	2.54	-	4.81
9	*β*-Lonone	1.02729	0.12	87.36	28.69
10	Eucalyptol	1.02719	4.6	4.75	-
11	Geraniol	1.02364	1.1	87.21	66.70
12	Linalool oxide A	1.01593	100	1.21	0.15
13	Hexanal	1.01413	5	1.87	0.30
14	Linalool	1.00567	50	40.89	2.38

Note: “-”: volatile compounds not detected.

## Data Availability

The original contributions presented in the study are included in the article/Supplementary Materials, further inquiries can be directed to the corresponding author.

## Supplementary Materials

The following supporting information can be downloaded at: https://www.mdpi.com/article/10.3390/foods15030596/s1, Table S1. Average retention times and average peak areas of target compounds. Table S2. Sensory evaluation data of LT and WT. Table S3: Volatile compounds identified in the aroma concentrate of LT, LF and WT.
