# Supplementary material for: Enhancement Effect of Lemon Flower on the Flavor Quality of White Tea and Its Formation Mechanism
Source: Foods. 2026 Feb 6;15(3):596. doi: 10.3390/foods15030596 (PMC12897128; doi:10.3390/foods15030596)
Supplement: Supplementary file 1 [file foods-15-00596-s001.zip › foods-4106304-supplementary.pdf]

Table S1. Average retention times and average peak areas of target compounds.

| Compounds | Average retention time (min) | Average peak area in LT | Average peak area in WT |
|---|---|---|---|
| EC | 6.633 | 177140 | 131432 |
| EGC | 7.713 | 896286 | 798291 |
| EGCG | 12.839 | 2013902 | 2132419 |
| C | 13.233 | 92061 | 91104 |
| ECG | 18.923 | 1230822 | 1434365 |
| GA | - | 474 | 523 |

Table S2. Sensory evaluation data of LT and WT.

| Compounds | LT | WT |
|---|---|---|
| Appearance | 86.1±4.2 | 85.2±6.7 |
| Soup | 88.4±2.6 | 87.2±4.1 |
| Aroma | 83.4±4.2 | 90.5±2.2 |
| Taste | 85.2±3.6 | 89.3±2.9 |
| Tea leaves residue | 91.2±5.6 | 90.1±4.3 |

Table S3. Volatile compounds identified in the aroma concentrate of LT, LF and WT.

| Category | Volatile compounds | CAS | Chemical formula | RT (min) | RI[a]/RI[b] | Content (μg/kg) | | |
|---|---|---|---|---|---|---|---|---|
| | | | | | | LT | LF | WT |
| Terpenoids | *α*-Thujene | 2867-05-2 | $C_{10}H_{16}$ | 5.453 | 1020/1028 | / | 9.90±3.68 | / |
| | *α*-Muurolene | 10208-80-7 | $C_{15}H_{24}$ | 36.468 | 1711/1726 | / | 5.58±1.16 | / |
| | *β*-Pinene | 127-91-3 | $C_{10}H_{16}$ | 7.449 | 1094/1112 | 4.82±0.73[b] | 103.07±19.93[a] | / |
| | 3-Carene | 13466-78-9 | $C_{10}H_{16}$ | 8.642 | 1132/1147 | / | 5.82±3.51 | / |
| | Limonene | 138-86-3 | $C_{10}H_{16}$ | 10.692 | 1175/1199 | 35.31±3.99[b] | 1816.77±336.43[a] | 26.81±1.21[b] |
| | *β*-Ocimene | 13877-91-3 | $C_{10}H_{16}$ | 12.773 | 1237/1250 | 5.84±0.55[b] | 473.45±21.47[a] | 7.00±1.19[b] |
| | Germacrene B | 15423-57-1 | $C_{15}H_{24}$ | 40.112 | 1808/1819 | / | 23.00±4.63 | / |
| | *α*-Bergamotene | 17699-05-7 | $C_{15}H_{24}$ | 29.187 | 1579/1584 | / | 155.94±7.82 | / |
| | (E)-*β*-Farnesene | 18794-84-8 | $C_{15}H_{24}$ | 34.088 | 1666/1665 | / | 33.19±3.48 | / |
| | δ-Elemene | 20307-84-0 | $C_{15}H_{24}$ | 22.634 | 1461/1470 | / | 29.66±3.45 | / |
| | Germacrene D | 23986-74-5 | $C_{15}H_{24}$ | 35.488 | 1691/1710 | / | 87.94±8.95 | / |
| | (+)-Bicyclogermacrene | 24703-35-3 | $C_{15}H_{24}$ | 36.656 | 1717/1735 | / | 77.42±8.84 | / |
| | *α*-(E)-Bisabolene | 25532-79-0 | $C_{15}H_{24}$ | 38.665 | 1768/1741 | / | 9.71±1.14 | / |
| | *γ*-Elemene | 29873-99-2 | $C_{15}H_{24}$ | 32.013 | 1626/1642 | / | 5.46±3.78 | / |
| | (E)-*β*-Ocimene | 3779-61-1 | $C_{10}H_{16}$ | 11.990 | 1221/1250 | 2.83±0.37[b] | 62.65±12.68[a] | / |
| | *β*-Bisabolene | 495-61-4 | $C_{15}H_{24}$ | 36.853 | 1722/1728 | / | 136.09±22.53 | / |
| | Terpinolene | 586-62-9 | $C_{10}H_{16}$ | 13.771 | 1275/1284 | 1.66±0.07[b] | 34.74±3.90[a] | / |
| | *α*-Humulene | 6753-98-6 | $C_{15}H_{24}$ | 33.201 | 1651/1667 | / | 41.89±5.01 | / |
| | *α*-Pinene | 80-56-8 | $C_{10}H_{16}$ | 5.308 | 1015/1027 | / | 28.87±9.91 | / |
| | Caryophyllene | 87-44-5 | $C_{15}H_{24}$ | 29.403 | 1584/1595 | / | 327.25±32.00 | / |
| | Thymol | 89-83-8 | $C_{10}H_{14}O$ | 49.792 | 2187/2189 | / | 32.01±4.96 | / |
| | *γ*-Terpinene | 99-85-4 | $C_{10}H_{16}$ | 12.291 | 1240/1246 | 6.97±0.88[b] | 317.40±22.81[a] | / |

| | | | | | | | | |
|---|---|---|---|---|---|---|---|---|
| | Total | | | | | 57.43±6.41$^{b}$ | 3817.83±400.57$^{a}$ | 33.81±1.58$^{b}$ |
| | Benzyl alcohol | 100-51-6 | $C_7H_8O$ | 42.085 | 1873/1870 | / | / | 28.63±3.41 |
| | (Z)-3-Nonen-1-ol | 10340-23-5 | $C_9H_{18}O$ | 35.406 | 1685/1682 | / | / | 2.41±0.60 |
| | Citronellol | 106-22-9 | $C_{10}H_{20}O$ | 38.949 | 1773/1765 | / | 22.80±2.06 | / |
| | Geraniol | 106-24-1 | $C_{10}H_{18}O$ | 41.498 | 1852/1847 | 18.55±4.16$^{b}$ | 33.15±3.49$^{b}$ | 172.04±25.36$^{a}$ |
| | Nerol | 106-25-2 | $C_{10}H_{18}O$ | 40.002 | 1800/1797 | 1.10±0.13$^{b}$ | 35.37±2.73$^{a}$ | 4.38±1.03$^{b}$ |
| | 1-Hexanol | 111-27-3 | $C_6H_{14}O$ | 17.820 | 1353/1355 | / | 2.08±0.63 | / |
| | $\alpha$-Santalol | 115-71-9 | $C_{15}H_{24}O$ | 32.438 | 1637/1681 | / | 6.94±0.65 | / |
| | 1-Nonanol | 143-08-8 | $C_9H_{20}O$ | 34.311 | 1665/1660 | / | / | 5.54±0.66 |
| | 1-Heneicosanol | 15594-90-8 | $C_{21}H_{44}O$ | 53.592 | 2384/2380 | / | 1.39±0.67 | / |
| | (E)-Geranylgeraniol | 24034-73-9 | $C_{20}H_{34}O$ | 50.374 | 2215/2201 | / | 9.53±2.65 | / |
| Alcohols | Nerolidol | 40716-66-3 | $C_{15}H_{26}O$ | 46.629 | 2043/2042 | 39.75±8.13$^{b}$ | 277.41±41.96$^{a}$ | 1.40±0.15$^{b}$ |
| | Farnesyl alcohol | 4602-84-0 | $C_{15}H_{26}O$ | 53.167 | 2361/2350 | / | 54.23±13.61 | / |
| | Eucalyptol | 470-82-6 | $C_{10}H_{18}O$ | 11.104 | 1205/1212 | 4.22±0.41$^{b}$ | 114.42±10.71$^{a}$ | / |
| | $\alpha$-Bisabolol | 515-69-5 | $C_{15}H_{26}O$ | 50.434 | 2218/2214 | / | 3.80±1.04 | / |
| | 4-Terpineol | 562-74-3 | $C_{10}H_{18}O$ | 30.419 | 1597/1602 | 3.55±0.19$^{a}$ | 16.92±5.95$^{a}$ | / |
| | Phenylethyl Alcohol | 60-12-8 | $C_8H_{10}O$ | 43.010 | 1905/1907 | 5.89±1.10$^{b}$ | / | 106.66±8.94$^{a}$ |
| | Spathulenol | 6750-60-3 | $C_{15}H_{24}O$ | 48.357 | 2120/2136 | / | 1.64±0.52 | / |
| | Linalool | 78-70-6 | $C_{10}H_{18}O$ | 27.916 | 1549/1547 | 395.30±26.53$^{b}$ | 476.45±47.48$^{a}$ | 279.17±6.17$^{c}$ |
| | (E)-2-Hexen-1-ol | 928-95-0 | $C_6H_{12}O$ | 20.382 | 1409/1406 | / | 6.01±0.34 | / |
| | (Z)-3-Hexenol | 928-96-1 | $C_6H_{12}O$ | 19.345 | 1390/1382 | / | / | 2.97±0.37 |
| | $\alpha$-Terpineol | 98-55-5 | $C_{10}H_{18}O$ | 35.878 | 1695/1697 | 8.21±0.6$^{b}$ | 55.74±5.09$^{a}$ | 3.92±1.46$^{b}$ |
| | Carveol | 99-48-9 | $C_{10}H_{1}O$ | 40.979 | 1835/1824 | / | 8.27±0.68 | / |
| | Total | | | | | 476.56±29.57$^{b}$ | 1126.14±119.85$^{a}$ | 607.13±18.06$^{b}$ |
| Aldehydes | Benzaldehyde | 100-52-7 | $C_7H_6O$ | 25.552 | 1507/1520 | 4.25±0.41$^{b}$ | 1.20±0.29$^{c}$ | 28.74±2.52$^{a}$ |

| | | | | | | | | |
|---|---|---|---|---|---|---|---|---|
| | Citronellal | 106-23-0 | $C_{10}H_{18}O$ | 23.507 | 1472/1478 | / | 10.42±2.40 | / |
| | Neral | 106-26-3 | $C_{10}H_{16}O$ | 34.647 | 1673/1680 | / | 107.93±15.45 | / |
| | 2-Butyl-2-octenal | 13019-16-4 | $C_{12}H_{22}O$ | 33.688 | 1653/1656 | / | / | 6.48±0.39 |
| | *α*-Citral | 141-27-5 | $C_{10}H_{16}O$ | 37.123 | 1726/1732 | / | 155.13±19.55 | / |
| | Farnesyl aldehyde | 502-67-0 | $C_{15}H_{24}O$ | 51.315 | 2263/2270 | / | 10.65±2.61 | / |
| | 2-Hexenal | 505-57-7 | $C_6H_{10}O$ | 11.709 | 1213/1213 | 1.53±0.26$^{b}$ | 29.32±5.51$^{a}$ | 4.07±0.4$^{b}$ |
| | Citral | 5392-40-5 | $C_{10}H_{16}O$ | 36.957 | 1719/1717 | / | / | 5.48±1.34 |
| | *β*-Sinensal | 60066-88-8 | $C_{15}H_{22}O$ | 50.560 | 2225/2238 | / | 3.44±1.77 | / |
| | Hexanal | 66-25-1 | $C_6H_{12}O$ | 6.972 | 1077/1083 | 1.81±0.23$^{b}$ | 11.62±1.48$^{a}$ | 3.50±0.14$^{b}$ |
| | 2-Methylbutyraldehyde | 96-17-3 | $C_5H_{10}O$ | 3.319 | 911/914 | / | / | 1.28±0.32 |
| | Total | | | | | 7.59±0.87$^{b}$ | 329.71±44.24$^{a}$ | 49.55±4.68$^{b}$ |
| Ketones | Methyl heptenone | 110-93-0 | $C_8H_{14}O$ | 16.473 | 1331/1339 | / | 4.42±0.41$^{a}$ | 4.17±0.41$^{a}$ |
| | 3,5-Octadien-2-one | 30086-02-3 | $C_8H_{12}O$ | 28.383 | 1558/1570 | / | / | 35.17±0.73 |
| | Geranylacetone | 3796-70-1 | $C_{13}H_{22}O$ | 41.421 | 1852/1859 | / | 3.90±1.21 | / |
| | Jasmone | 488-10-8 | $C_{11}H_{16}O$ | 43.644 | 1931/1961 | 4.52±0.54$^{a}$ | / | 4.92±0.8a |
| | (+)-Dihydrocarvone | 5524-05-0 | $C_{10}H_{16}O$ | 31.134 | 1610/1613 | / | 4.07±0.66 | / |
| | *β*-Lonone | 79-77-6 | $C_{13}H_{20}O$ | 43.519 | 1924/1941 | 2.03±0.2$^{b}$ | / | 8.07±0.59$^{a}$ |
| | Acetophenone | 98-86-2 | $C_8H_8O$ | 32.371 | 1632/1636 | / | / | 1.08±0.27 |
| | Total | | | | | 6.55±0.71$^{c}$ | 12.39±2.07$^{b}$ | 53.42±2.33$^{a}$ |
| Esters | Geranyl acetate | 105-87-3 | $C_{12}H_{20}O_2$ | 38.300 | 1758/1752 | / | 15.42±8.47 | / |
| | Methyl salicylate | 119-36-8 | $C_8H_8O_3$ | 38.301 | 1758/1765 | 18.25±2.7$^{b}$ | / | 167.18±9.24$^{a}$ |
| | Methyl anthranilate | 134-20-3 | $C_8H_9NO_2$ | 50.716 | 2233/2232 | 58.00±5.89$^{b}$ | 86.54±8.29$^{a}$ | / |
| | Total | | | | | 76.25±3.68$^{c}$ | 101.96±7.81$^{b}$ | 167.18±9.24$^{a}$ |
| Heterocyclic | Caryophyllene oxide | 1139-30-6 | $C_{15}H_{24}O$ | 44.554 | 1965/1989 | / | 7.09±1.9 | / |

| | | | | | | | | |
|---|---|---|---|---|---|---|---|---|
| compounds | p-cymenene | 1195-32-0 | $C_{10}H_{12}$ | 21.041 | 1427/1444 | / | 6.19±1.01 | / |
| | Indole | 120-72-9 | $C_8H_7N$ | 54.581 | 2441/2445 | 2.59±0.36[b] | 150.40±18.7[a] | / |
| | 2-Ethylfuran | 3208-16-0 | $C_6H_8O$ | 3.964 | 948/951 | / | / | 2.02±0.17 |
| | Linalool oxide A | 34995-77-2 | $C_{10}H_{18}O_2$ | 23.462 | 1467/1452 | 23.32±1.06[b] | / | 35.38±2.38[a] |
| | 2-Pentylfuran | 3777-69-3 | $C_9H_{14}O$ | 11.398 | 1202/1232 | 3.40±0.36[b] | / | 15.87±9.08[a] |
| | Linalool oxide B | 5989-33-3 | $C_{10}H_{18}O_2$ | 21.959 | 1439/1444 | 9.42±0.57[b] | / | 20.6±1.92[a] |
| | Linalool oxide D | 14009-71-3 | $C_{10}H_{18}O_2$ | 38.762 | 1768/1751 | 7.68±0.51[a] | / | 8.91±0.74[a] |
| | (E)-Limonene oxide | 6909-30-4 | $C_{10}H_{16}O$ | 22.201 | 1447/1470 | / | 25.93±3.77 | / |
| | p-Cymene | 99-87-6 | $C_{10}H_{14}$ | 13.349 | 1263/1272 | 9.47±0.43[b] | 96.35±8.39[a] | / |
| | Total | | | | | 55.89±1.75[b] | 285.95±26.79[a] | 82.78±5.34[b] |
| Others | Styrene | 100-42-5 | $C_8H_8$ | 13.064 | 1244/1261 | 3.29±0.37 | / | / |
| | 1,3-(E)-5-(Z)-Octatriene | 40087-61-4 | $C_8H_{12}$ | 7.463 | 1094/1108 | / | / | 1.99±0.17 |
| | (E,E)-cosmene | 460-01-5 | $C_{10}H_{14}$ | 21.611 | 1439/1460 | / | 6.80±1.74 | / |
| | Total | | | | | 3.29±0.37[b] | 6.80±1.74[a] | 1.99±0.17[b] |

RT, retention time.

[a], Retention index of compounds on DB-WAX.

[b], Retention index of compounds in references.
